# Leber Hereditary Optic Neuropathy: Case Report and Literature Review

**DOI:** 10.7759/cureus.7745

**Published:** 2020-04-20

**Authors:** Asia Filatov, Javed L Khanni, Patricio S Espinosa

**Affiliations:** 1 Neurology, Charles E. Schmidt College of Medicine, Florida Atlantic University, Boca Raton, USA; 2 Neurology, Marcus Neuroscience Institute, Boca Raton Regional Hospital, Boca Raton, USA

**Keywords:** leber hereditary optic neuropathy, optic neuropathy, mitochondrial dna mutation, vision loss, glaucoma, lhon

## Abstract

Leber hereditary optic neuropathy (LHON) is a genetic condition that typically presents with unilateral, painless, sub-acute central vision loss followed by contralateral vision loss after a few weeks to months. It is a rare disease that typically affects young adults - men more than women - and is a relatively common cause of blindness. It is due to a mutation in mitochondrial DNA (mtDNA). The majority (more than 95%) of patients have one of three mtDNA point mutations: m.14484T→C, m.3460G→A, or m.11778G→A. These mutations lead to disruption of the mitochondrial respiratory chain activating pro-apoptotic pathways. For reasons unknown, this insult tends to affect the retinal ganglion cells more than any other cell in the body, leading to the disease state. Due to its low prevalence in the United States (1:50,000), this diagnosis is often overlooked, misdiagnosed, and mismanaged, which may exacerbate symptoms. It is essential then for physicians to recognize the presentation of and understand the diagnostic work-up for LHON. In this case report, we present the diagnostic challenges of a patient who presented with progressive vision loss, discuss the various differential diagnoses, review the literature on LHON, and propose an explanatory model for vision loss in patients with LHON.

## Introduction

Leber hereditary optic neuropathy (LHON) is a genetic disorder that primarily involves mitochondrial DNA (mtDNA), leading to mitochondrion dysfunction in the eye causing focal degeneration of the retinal ganglion cell (RGC) layer in the retina and of the optic nerve. This is associated with bilateral, acute-to-sub-acute, severe central vision loss, which normally presents in one’s twenties and thirties, affecting more men than women [1]. The loss of vision typically starts in one eye followed by contralateral vision loss after weeks or months. Up to 50% of affected patients do not have a family history of vision loss. Prognosis of the vision loss is poor, and most of those living with the condition remain significantly visually impaired. More than 9 in 10 patients with LHON suffer from either of three obvious causative mtDNA mutations: m.14484T→C, m.3460G→A, or m.11778G→A [2]. Another intriguing aspect of LHON genetics is that the associated mutations have incomplete penetrance. This means that not everyone with one of these three mutations develops LHON despite the fact that more than 90% of patients with LHON have one of the aforementioned mutations. Clearly, mtDNA mutation is not enough by itself to cause LHON. To that end, secondary modulators such as factors related to nuclear genetics and the environment have been considered contributors [3]. This case study highlights the difficulty in diagnosing LHON and how it may masquerade as other conditions if careful clinical analysis is not conducted, and discusses a possible trigger for LHON in patients with one of the three mutations.

## Case presentation

A 49-year-old Jamaican male presented with a recent history of vision loss. About three or four months prior to his hospitalization, he suffered sudden-onset non-progressive decrease in visual acuity of the left eye. Later that day, he experienced decreased vision in his right eye followed by worsening vision of the left eye two days later. Approximately one week later, he developed numbness in the plantar surface of his right foot and right calf followed by similar symptoms in the left leg two weeks later. He was initially followed in the community as an outpatient and was prescribed high-dose steroids for a suspected diagnosis of neuromyelitis optica (NMO). The patient was also evaluated by an ophthalmologist who found increased intraocular pressure and diagnosed him with glaucoma in the left eye. As symptoms progressed despite outpatient treatment, he sought care at the Marcus Neuroscience Institute at Boca Raton Regional Hospital for further evaluation and management. He had no other significant medical or family history of vision loss.

**Figure 1:**
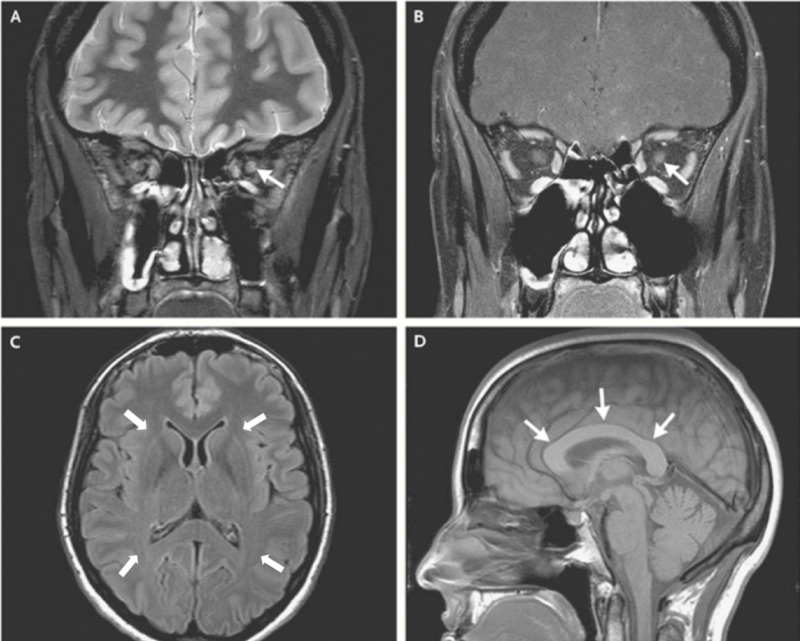
MRI of the Orbits and the Head. (A) Coronal, T2-weighted, high-resolution, short-tau-inversion-recovery MRI indicating an increased signal in the left optic nerve (white arrow). (B) Coronal, contrast-enhanced, high-resolution, fat-suppressed, T1-weighted image indicating mild hazy-enhancement of the left optic nerve (white arrow). (C) Axial, fluid-attenuated, inversion recovery imaging indicates no abnormal lesions of the white matter. (D) Sagittal, nonconstrast-enhanced, T1-weighted image indicating no lesions in the corpus callosum, which is normal in caliber and shape (white arrows). MRI, magnetic resonance imaging

In the Emergency Department, he was hemodynamically stable, with visual acuity of 20/200 in the left eye and 20/70 in the right eye. Confrontational visual field testing showed a left upper quadrant deficit in the left eye, with red dyschromatopsia and increased cup-to-disc ratio doubled that of the right eye. Optokinetic flag testing was normal bilaterally. Computed tomography (CT) scan of the head was unremarkable. A repeat magnetic resonance imaging (MRI) of the brain, orbits, and C-spine was negative for demyelinating lesions, as seen in NMO (Figure 1). A lumbar puncture was performed with cerebrospinal fluid (CSF) analysis showing no oligoclonal banding at a dilution factor of 46, negative immunoglobulin G (IgG) index (indicating no intracerebral IgG production), and negative cytology for viral or bacterial etiology (Table 1). An inpatient ophthalmology consultation was obtained, which demonstrated no evidence of optic neuritis and confirmed the diagnosis of glaucoma.

**Table 1:** CSF Analysis CSF, cerebrospinal fluid

Variable	Reference Range [4]	Test
Glucose (fasting) (mg/dL)	50-75	84
Total protein (mg/dL)	5-55	46
Color	Colorless	Colorless
Turbidity	Clear	Clear
Nucleated cell count (per mm^3^)	0-5	3
Red blood cell count (per mm^3^)	0-5	1
Differential count (%)		
Blasts	0	0
Bands	0	0
Neutrophils	0	12
Lymphocytes	0–100	14
Reactive or atypical lymphocytes	0	0
Monocytes	0–100	67
Eosinophils	0	0
Basophils	0	0
Macrophages	0	0
Xanthochromia	None	None
Venereal Disease Research Laboratory test	Nonreactive	Nonreactive
Oligoclonal bands	No banding seen in CSF concentrated by a factor of 80	No banding seen in CSF concentrated by a factor of 56
Microbiology	No microscopic, antibody, or biochemical detection	No viruses, fungi, or bacteria detected in CSF

The patient was discharged and instructed to follow up with a neuro-ophthalmologist. On examination, corrected acuity was 20/200 in both eyes. Color vision was 8/8 in the right eye and 7/8 in the left eye. There was a left afferent pupillary defect. On fundoscopic examination, the optic nerve was sharp in both eyes, with mild-to-moderate temporal pallor in the right eye and severe temporal pallor in the left eye. The cup-to-disc ratio was 0.5 in the right eye and 0.8 in the left eye. There was a central scotoma in both eyes. More specifically, the left eye was found to have a superior field cut by Amsler grid, a type of central scotoma that is consistent with optic neuropathy. Based on these findings, negative neuroimaging, and normal CSF analysis, our patient was recommended to undergo genetic testing for LHON. It yielded a homoplasmic, m.11778G→A LHON mutation.

Differential diagnoses

Neuromyelitis Optica Spectrum Disorder

NMO is an inflammatory disorder of the central nervous system, which, in most cases, presents as optic myelitis and/or neuritis. Our patient was negative for autoantibody to aquaporin 4, eliminating NMO from diagnostic consideration [5].

Muscular Dystrophy

He had no signs of muscle weakness or wasting, respiratory insufficiency, or cardiac dysfunction that is usually seen in muscular dystrophy [6].

Infection

Lyme disease, mumps, measles, cat-scratch fever, syphilis, and herpes may cause optic neuritis [7]. CSF analysis ruled-out viral, bacterial, or fungal infection.

Sarcoidosis

Sarcoidosis causes symptoms such as lacrimation, red eyes, blurred vision, reddish bumps on the skin, kidney stone formation, swollen and painful joints, hepatomegaly, and nervous system effects. Nervous system involvement includes meningitis, hearing loss, seizures, depression, and dementia [7]. Our patient experienced numbness on the bottom of the right foot and calf but no joint pain. The lack of multi-organ involvement ruled out sarcoidosis [8].

Isolated Optic Neuritis

Optic neuritis is an immune-mediated neurologic disorder that results in demyelination and inflammation of the optic nerve. It may be isolated or be a symptom of multiple sclerosis or NMO. The absence of pain on presentation and negative imaging precludes this diagnosis. [7]. The absence of oligoclonal bands (Table 1) and negative neuroimaging ruled out multiple sclerosis [9].

Leber Hereditary Optic Neuropathy

LHON is known to cause subacute, painless, severe loss of vision in one eye, and it progresses to loss of vision in a similar fashion as the other eye [7]. The patient’s symptoms began with loss of vision in his left eye and progressed to involve the right eye. This diagnosis confirmed by detection of m.11778G→A mutation on genetic analysis.

## Discussion

LHON is mainly associated with painless, subacute loss of central vision, which is bilateral and mostly occurs in young adult males. Carriers of any one of the three mutations linked to LHON experience significant vision loss before they reach the fifth decade of life. This has been observed in multiple studies, including various populations from around the world [10]. Our patient exemplifies the typical disease course of individuals with LHON. However, the question remains: what is the trigger for vision loss in this otherwise healthy male 11778G→A mutation carrier? We propose that the most likely explanation is that raised intraocular pressure triggered LHON conversion from dormant to active disease, leading to vision loss. Mitochondria frequently crowd the optic nerve as the ganglion cell dives into the optic nerve from the posterior retina. The increased intraocular pressure was an added stress on the genetically susceptible organelle, leading to organelle failure. Moreover, his glaucomatous changes may have been exacerbated by steroids that were given for the treatment of presumptive NMO. The drastic deterioration of vision that appeared suddenly rather than gradually involving both eyes may be a result of expedited RGC loss that typically occurs in end-stage disease. Given that the mechanism for vison loss in both LHON and glaucoma involves loss of RGC, it is reasonable to postulate that our patient’s sudden, acute, bilateral vision loss was due to synergistically, deleterious effects of glaucoma and LHON.

The process by which raised intraocular pressure led to this patient’s vision loss is multifactorial. Biologically, the precarious homeostatic state that prevails inside the RGCs could have been perturbed by a buildup of pathogens related to LHON mutations. Increased intraocular pressures may have exacerbated these perturbations by decreasing axoplasmic flow or impairing the optic nerve head vascular supply at the critical transition region spanning the lamina cribrosa [11]. In LHON, parvocellular RGCs (P-cells) are lost to a greater extent than magnocellular RGCs (M-cells). The P-cells in papillomacular bundles possess a smaller cross-section area compared to the relatively large M-cells. This anatomical fact may be responsible for the susceptibility to increased pressure on axoplasmic flow, particularly in the high energy-dependent unmyelinated segment of the optic nerve. Here, mitochondria are more affected in the axons of RGCs due to raised intraocular pressure. These damaged mitochondria, in combination with mutated mtDNA, will significantly reduce mitochondrial respiratory chain efficiency and result in the formation and release of pro-apoptotic cytochrome c molecules [12]. The final result is sudden, acute, non-progressive vision loss, as seen in our patient.

## Conclusions

LHON is a multifactorial disease primarily associated with mtDNA mutations; however, these mutations are not sufficient alone to result in loss of sight. Secondary factors likely contributed to LHON conversion to symptomatic disease. LHON is a difficult disease to diagnose. Its presentation may mimic numerous other disorders such as NMO, isolated optic neuritis, and sarcoidosis. Making the diagnosis requires a high clinical suspicion in the appropriate clinical setting. It requires multiple resources in equipment and manpower for diagnosis. Identification of specific mutations in the mtDNA is the gold standard for diagnosis. Unfortunately, there are no current FDA-approved medications for the disease. Therefore, LHON patients need to be provided with supportive therapy. This case report postulates a possible trigger leading to symptomatic LHON and adds to the growing body of literature on LHON.
